# Towards Explainable Graph Embeddings for Gait Assessment Using Per-Cluster Dimensional Weighting

**DOI:** 10.3390/s25134106

**Published:** 2025-06-30

**Authors:** Chris Lochhead, Robert B. Fisher

**Affiliations:** University of Edinburgh, Old College, South Bridge, Edinburgh EH8 9YL, UK; rbf@ed.ac.uk

**Keywords:** gait assessment, computer vision, graph networks, older adult care

## Abstract

As gaitpathology assessment systems improve both in accuracy and efficiency, the prospect of using these systems in real healthcare applications is becoming more realistic. Although gait analysis systems have proven capable of detecting gait abnormalities in supervised tasks in laboratories and clinics, there is comparatively little investigation into making such systems explainable to healthcare professionals who would use gait analysis in practice in home-based settings. There is a “black box” problem with existing machine learning models, where healthcare professionals are expected to “trust” the model making diagnoses without understanding its underlying reasoning. To address this applicational barrier, an end-to-end pipeline is introduced here for creating graph feature embeddings, generated using a bespoke Spatio-temporal Graph Convolutional Network and per-joint Principal Component Analysis. The latent graph embeddings produced by this framework led to a novel semi-supervised weighting function which quantifies and ranks the most important joint features, which are used to provide a description for each pathology. Using these embeddings with a K-means clustering approach, the proposed method also outperforms the state of the art by between 4.53 and 16% in classification accuracy across three datasets with a total of 14 different simulated gait pathologies from minor limping to ataxic gait. The resulting system provides a workable improvement to at-home gait assessment applications by providing accurate and explainable descriptions of the nature of detected gait abnormalities without need of prior labeled descriptions of detected pathologies.

## 1. Introduction

Gait assessment using computer vision is a demonstrably effective method for detecting gait abnormalities, particularly in older adults or people affected by age-related diseases [1,2,3,4]. Beyond the relatively simpler problems of person recognition or fall detection which have been developed to clinically high accuracy [5,6,7], the attention of researchers has turned to the more complex task of gait abnormality assessment. Gait assessment is defined as the task of identifying changes in the gait of an individual or similar changes across individuals (i.e., the presence of a limp) in contrast to gait recognition, which concerns person identification using only gait data. This problem domain is useful for the detection and monitoring of diseases [8,9,10], the assessment of at-home safety [11,12] and the quantification of health attributes like patient stability and balance [13,14].

There are two core gaps in gait assessment that act as a barrier between research and real world use: (1) the lack of research on unsupervised gait assessment problems and (2) the lack of explainability in gait assessment models. Research focusing on gait assessment tends to use supervised tasks with labeled datasets featuring distinct gait pathologies to train their models [3,15,16], but in application—for example, in an at-home gait monitoring system—these models are likely to only have access to examples of a person’s current gait. Explainability is vital in this domain, as low acceptance in application is often attributed to be a result of lack of interpretability by users [17,18,19].

This work proposes an ensemble approach using trained Graph Convolutional Networks and Principal Component Analysis (PCA) for producing highly descriptive feature embeddings which are more readily separable using K-means clustering than regular gait graph data. GCN-based architectures are extremely useful in this domain for representing gait as graphs are typically the superior method in domains where laboratory conditions are not used for data recording and accurate joint key-point extraction is difficult. Dimensionality reduction methods such as PCA then have utility in simplifying these gait graphs into more explainable representations. Using these embeddings, a novel weighted feature importance score is developed. This score quantifies the importance of different body key-points for distinguishing between a cluster representing a pathology and the cluster representing a patient’s “regular” gait. This score is calculated using a semi-supervised paradigm, in which the method is only aware of which cluster represents “normal” gait, with the other clusters remaining unlabeled and without explicit context of the type of pathologies they exhibit. This novel scoring system provides vital explainability to a model’s prediction of gait by illustrating the model’s decisions by its observations of each body part.

To achieve the goal of developing a more explainable gait assessment system with potential real-life applications in healthcare, two main contributions are proposed. First, a novel ensemble approach using trained ST-TAGCN embeddings with context-aware PCA and K-means is proposed. Second, a novel semi-supervised feature importance score “DimWise” is introduced, capable of ranking the relative importance of body joints to provide an explainable description of clusters without requiring prior knowledge of abnormal gait pathologies. This scoring metric allows for the description via joint-ranking any gait pathology. In practice this means that any novel deviations in a user’s gait can be described without explicit labels, and communicated in a manner that is explainable to healthcare professionals who would use said data to inform their care decisions.

The research question for this work can be summarized as: How can an explainable machine learning framework be developed to both more effectively cluster gait pathologies and be used to describe gait pathology clusters in an interpretable way? To answer this question, the following contributions are introduced:

(1) A novel ST-GCN-based embedding approach, supplemented with context-aware PCA to generate descriptive single-frame gait representations for more accurate clustering. These embeddings achieve state-of-the-art accuracy and f1 scores in a supervised gait abnormality assessment when used with K-means.
(2) A novel feature importance score named DimWise, capable of ranking, per-cluster, the importance of features to provide an explainable description of clusters without requiring prior knowledge of the pathologies in a dataset other than what the “regular” gait class is.
(3) A pair of confidence and severity scores to communicate to a user both the confidence the system has for its prediction and the estimated degree of difference of a pathology from an individual’s regular gait.

After an overview of the current state of at-home gait analysis systems and an investigation into semi-supervised gait assessment, machine learning (ML) powered embedding systems and K-means feature importance algorithms (see Section 2), a description of the novel embedding and feature importance calculation system is provided (see Section 3). Additionally provided is an overview of the datasets and pre-processing methodology used prior to the implementation of the novel clustering system. In Section 4 there is an overview of accuracy comparisons with the current state of the art on three simulated gait pathology datasets on the supervised assessment task (achieving between 4.53 and 15% accuracy improvement), alongside a discussion of the interpretability and explainability of the resulting feature scores. To conclude, limitations, such as the types of prior information about gait pathology classes that are still required for this methodology are given, as well as a discussion of future work to build on this new direction of research (see Section 6).

## 2. Related Work

Machine learning (ML) for gait assessment is a broad field of work, encompassing simple methods such as K-means or Logistic Regression, to advanced neural network-based methods utilizing Graph Convolutional Networks and associated architectures. The most recent ML-powered gait assessment research is based on the ST-GCN. Originally developed in [20] for traffic control modeling, it has since been extensively used in gait recognition and assessment applications [4,10,16,21], assessing a wide variety of pathologies, such as freezing of gait events typical to Parkinson’s Disease [22] and general dementia-based illnesses [23], as well as specific simulated gait abnormalities such as gait asymmetry [24] and more complex abnormalities such as ataxic or lurching gait [25].

### 2.1. Machine Learning in Gait Assessment

The ST-GCN is the implementation of a convolutional network for graph-based data, in which the input stream is convolved both along the spatial dimension and then the temporal dimension of the input data in different combinations and orders depending on the specific implementation. The spatio-temporal gait data using these models are typically representations of individual body joints in space. These can be as simple as joint co-ordinates to more second-order data derived from these co-ordinates, namely joint velocities or bone angle vectors. Much of the recent gait assessment literature employs networks made up of these ST-GCN blocks, for example [26], who use a network made up of nine sequential ST-GCN blocks along with a three-stream input paradigm, feeding their network separate streams for joint position, velocity and bone angles simultaneously to achieve up to 90% person recognition accuracy on the CASIA-B dataset [27].

Beyond person recognition, researchers in [21] employ a two-stream ST-GCN, using bone and joint features as inputs for the task of Parkinsonian gait severity assessment, achieving 65.7% mean accuracy across the six classes of degradation, indicating the feasibility of using gait to distinguish between different stages of age-related disease. Researchers in [16] use another three-stream approach along with selective joint aggregation, early model fusion and a spatial dimension attention module to obtain 92.6% mean accuracy across two simulated gait pathology datasets (*n* = 22), demonstrating the feasibility of using ML models to accurately distinguish between distinct pathologies.

Ref. [28] develops an ST-GCN variant employing temporal attention, using short-form gait instances segmented by gait cycle and using joint velocities as the only input stream. Achieving 94.38% on a complex simulated gait pathology dataset (*n* = 15), they demonstrate that gait assessment can be trained at a far lower computational cost than previous research (approximately 100 times faster per training epoch than the second best model [16]), highlighting the feasibility of real-world use in at-home systems. These results also demonstrate that ST-GCN model architectures can be modified such that different types of graphical input data perform more optimally. In summary, modern ML and particularly GCN-based approaches to gait abnormality assessment consistently achieve a >90% accuracy and f1 score on a variety of benchmarks; however, these benchmarks are typically datasets recorded in laboratory conditions as opposed to a more realistic “in-the-wild” approach.

### 2.2. Gait Pathology Datasets

Simulated gait datasets which are publicly available are typically recorded and distributed using accelerometer or gyroscopic data instead of image-based methods. As a result, there is a noted lack of open-source gait datasets where entire gait skeletons can be reliably extracted.

In [24,25], gait datasets are introduced to specifically simulate age-related gait abnormalities (*n* = 10) and more general gait asymmetries (*n* = 10), respectively. The former dataset exhibits a total of five complex pathological gait patterns such as antalgic and ataxic gait, acted out by healthy participants following a series of instructions. The latter dataset has a total of eight abnormalities intended to be subtle and to cause slight asymmetries in gait rather than specific complex gait symptoms. This is achieved by attaching either small weights to the ankles or placing raised insoles in one or both shoes to modify gait without the need for acting.

The Walking Gait dataset introduced in [15] follows the trend of the latter, developing a dataset of subtle gait asymmetries by modifying the shoes or attaching ankle weights to participants. In the WeightGait dataset [28], a mixture of these paradigms are employed, developing a dataset including both benign abnormalities like limps, and also instances of gait freezing and shuffling more akin to Parkinsonian gait. Furthermore, this dataset introduces overlapping gait abnormalities, rather than treating each abnormality as a distinct class. See Table A3 which contains the specific gait abnormalities present in the three datasets used in this research.

Due to the inherent privacy and ethical implications that accompany producing vision-based gait datasets, the vast majority of research that creates gait abnormality datasets either **(a)** do not make their video-based datasets open source as they contain vulnerable adults [23], **(b)** create their datasets without using a visual medium such as accelerometer data or pressure sensors [22] or **(c)** publish vision-based datasets of healthy adults simulating gait abnormalities to circumvent ethical concerns [24,25].

### 2.3. Unsupervised Gait Assessment and Embedding Methods

Generalized methods of machine learning model explainability exist, such as SHAP [29] and LIME [30]. SHAP estimates importance values by conducting extensive ablations on the data, recalculating model performance after removing every combination of input features. This exhaustive search method is computationally expensive and, hence, impractical for a method which would see use at scale in a healthcare setting. This domain would benefit from requiring fewer financial resources for processing data per patient and using processing that could be carried out on the edge (for example, in an at-home gait monitoring application).

LIME, on the other hand, is a computationally simpler model where an approximate model is estimated for each prediction and perturbed examples are assessed on that model to estimate feature importance on classification. DimWise, however, does not rely on the generation of any new data via perturbation, thus keeping it computationally less complex. LIME is also prone to unstable results depending on the perturbation strategy and is also constrained by the model selected for approximation. Typically, these are simple models with linear biases such as Logistic Regression, making the method less effective for non-linear data such as gait graphs.

To estimate feature importance after K-means clustering, researchers in [31] used decision trees to group input data in each cluster by feature to accurately describe each K-means cluster by communicating the most prominent value ranges for each feature per cluster. Building on this premise, Ref. [32] provides an algorithm using a penalty term to favor the synthesis of shallow decision trees to produce explainable clusters with a limited number of attributes describing each cluster.

While the majority of the gait assessment literature relies on labeled datasets and supervised learning methods, some research investigates unsupervised approaches, typically for the purpose of generating more explainable results. In [33], researchers use PCA to generate highly descriptive clusters from time-series knee flexion data to assess post-operative mobility improvement in sufferers of osteoarthritis. From these PCA embeddings, they were directly able to measure statistically distinct improvements in various gait attributes after surgery such as flexion angle and moment.

Researchers in [34] use an autoencoder framework to generate highly descriptive gait embeddings for multi-view person re-identification, achieving state-of-the-art re-identification accuracy and entanglement loss on the CASIA-B dataset. Ref [35] uses a multi-granularity network to produce a per-instance feature representation, achieving 96.6% rank 1 re-identification accuracy.

GCN architectures have a precedent for being used to generate feature embeddings for downstream tasks. [36,37], while autoencoders are a popular ML method for generating descriptive feature embeddings of high-dimensional data. This method lacks explainability because the latent embedding vector values have no explicit relation to any specific input values. GCN architectures are not typically fully connected, meaning each node at layer *l* predominantly describes the same node at layer l−1, combined with varying levels of context from its connected neighbors depending on the size of the node’s receptive field and the convolutional operation being performed (attention-based, weighted, mean, max, etc.). Deeper GCN models further limit explainability, as the receptive field at any given node expands with every additional layer and the influence of the original input for the node is diluted with more global context.

Models in the domain of unsupervised assessment exhibit limited performance and evaluation; for example, none of the reviewed literature evaluated the effectiveness of their models for explainability using domain experts such as healthcare professionals. This illustrates a potent gap in gait assessment research in that unsupervised approaches are not only under-researched, but also the quality of both the performance and evaluation of explainable methods require considerable improvement to reach a quality suitable for clinical use.

## 3. Methodology

### 3.1. Datasets and Pre-Processing

Three gait datasets with simulated pathologies serve as benchmarks to test the effectiveness and interpretability of the models across a variety of simulated gait conditions. They provide a broad range of gait pathologies across a total of 35 participants (15, 10 and 10, respectively) using different methods of abnormality generation ranging from acting, attachable weights, shoe implants and environmental obstacles. These three gait datasets are all of the datasets which exhibit synthetic gait abnormalities as classes, are published as gait skeleton data and are open source. A comprehensive overview of the datasets and their attributes is provided in Table 1.

As these open-source datasets exist in different formats, with different numbers of joint co-ordinates per skeleton ranging from 18 to 25, it is necessary to standardize the data so all three datasets are interpretable by one model architecture. To this end, all datasets were reformatted to fit the WeightGait dataset format in [28] which contains the least joints (*n* = 18): excluding fingers, toes and other likely redundant information as indicated in the experiments in [16]. Their ablations found that a single joint for each foot and hand instead of individual fingers and toes provided comparable performance and reduced unnecessary variance. All gait samples were reformatted from individual instances of a single skeleton to 7-frame gait cycle instances connected into a single spatio-temporal graph, with the joint *i* at frame *t* being connected to joint *i* at frames t+1 and t−1. Seven frames was observed to be the median gait cycle length in [28] and creating input graphs of this depth as opposed to entire gait videos of 150–200 frames [16] further reduces unhelpful variance in the data and contributes to a significant efficiency boost by reducing the number of channels necessary in the GCN model layers from 150 to 7.

**Figure 1:**
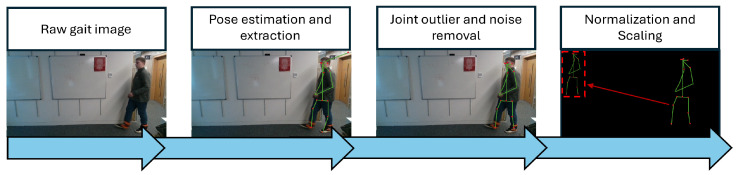
Pre-processing illustration for the WeightGait dataset. All steps except for joint outlier and noise removal had already been performed in the Pathological [25] and Shoe datasets [15] prior to release. Joint outlier and noise removal refers to bespoke functions for normalizing depth values from occlusion and resetting joints that are unnaturally far from their connected key points. Normalization and scaling refers to a series of operations which standardize the sizes and shapes of gait skeletons to ensure similar heights and limb lengths to de-emphasize person variation within gait abnormalities.

The pre-processing format in [28] (see Figure 1) was followed on each dataset where feasible. This was followed exactly for the WeightGait dataset; however, for the latter two datasets, all the steps except for joint outlier removal, noise removal and skeleton normalization were skipped as these operations had already been performed by the original researchers prior to publishing their datasets.

### 3.2. Machine Learning Model Embedding

Benchmarking reported in [9,28,38] indicates that graph-based gait sequence data is too complex and non-linear for non-deep learning methods such as K-means and Logistic Regression to perform adequately (see the results in Table 2 for our own benchmark of these methods).

**Figure 2:**
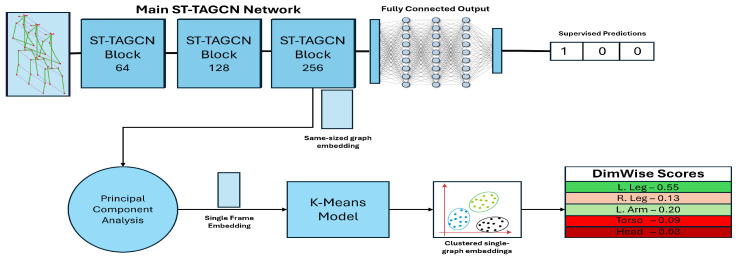
End-to-end diagram of the model used in this work. Three stacked ST-TAGCN modules feed their output into a fully connected network for the supervised task. Latent embeddings are taken from the last ST-TAGCN block and passed through a context-aware PCA to produce highly descriptive and compact single-frame gait embeddings. K-means clustering exposes clusters with different behaviors.

The ST-TAGCN network used in this work receives as input gait graphs of 18 nodes representing the human body as a set of $\left(v_{x},v_{y},v_{z}\right)$ 3D joint velocities. These 18-point gait graphs are temporally connected (i.e., the nose joint at frame *t* is connected to the nose joint at frame t+1) to gait graphs of 7 sequential frames representing a gait cycle, for an input graph shape of 18×7 nodes. The top layer of Figure 2 is initially trained with the one-hot encoding of the *n* gait anomaly classes as the output (see Section 4 for specific details of the model training method).

An ST-TAGCN model is trained using a 70:20:10 train–validation–test split of each of the three datasets for 120 epochs at a learning rate of 0.001 and using mini-batches of 128 according to the architecture given in Figure 2. The n×1 output vector is processed via Softmax (where *n* is the number of classes of the dataset being used) to provide a prediction, using the cross-entropy loss function. Stochastic gradient descent was the optimizer and the trained models were run across 5 folds for cross-validation to produce the results in Table 2.

To obtain the gait cycle embeddings, the fully-connected layers of the trained ST-TAGCN model are removed, with the final ST-TAGCN module’s output being reshaped from its typical 256×7 to the same shape as the input graph of 18×7 3D nodes.

To convert the graph data into a linear vector usable in a K-means model and process the data to accommodate greater interpretability, the 18×7 ST-TAGCN 3D output is converted into an 18 × 1 3D output, producing an embedding the equivalent size and shape of a single frame. It is hypothesized that the relatively shallow (3 modules) ST-TAGCN network imbues the embedded features with local regional context without sacrificing the dominant relation to its original input equivalent, as indicated by the DimWise results in Table 3 (see Section 4 for a more in-depth analysis). To achieve an 18×1 3D representation, PCA was employed individually for each of the 18 joints on the 1×7 3D temporal dimension, using the first principle component to reduce the number of 3D components per joint to one to produce an 18×1 embedding where each joint is effectively an embedding of that joint’s behavior across the entire gait cycle. Note that because of the three ST-GCN layers, the 18×1 vector does not have an exact correspondence with the 18 joints.

Using only 1 principle component was chosen as the first component would describe > 50% of the variance per group during testing and using one component likewise simplifies interpretability for downstream tasks, for example, clustering. To determine clustering accuracy, all gait instance embeddings are clustered according to pathology without supervision, using the existing gait instance labels to evaluate the clustering accuracy.

### 3.3. DimWise, Severity and Confidence Metrics

The DimWise score represents the importance of a body joint (feature) *f* for cluster *a* (denoted as $I_{f}^{a}$), calculated for each of the 18 features in the 18×1 PCA embedding. Intuitively, the score is designed to account for the variability of feature *f* in each cluster, the degree of overlap of each cluster and the degree of separation between clusters. The DimWise score is defined as:

(1) $$I_{f}^{a}=\frac{1}{\sigma_{f}^{2}}\cdot \left(1+O_{f}^{ar}\right)\cdot \left(1+|\mu_{f}^{r}-\mu_{f}^{a}|\right)$$

The first component $\frac{1}{\sigma_{f}^{2}}$ gives the inverse variance of feature *f* across all instances within cluster *a*. Refs. [39,40] reason that a lower variance of a feature within a cluster indicates that this feature is consistent and thereby predictably belongs to a certain cluster. Following this premise, a higher inverse variance correlates to a higher importance value that feature *f* has on the structure of cluster *a*.

$\left(1+O_{f}^{ar}\right)$ is the positively constrained overlap percentage between the instances of abnormal cluster *a* and cluster *r*, where cluster *r* is the cluster of the “regular” gait data for the dataset. This makes the DimWise metric semi-supervised in the sense that only the cluster representing “regular” gait must be known to perform these calculations. $O_{f}^{ar}$ is a measure of how many cluster instances are closer to the centroid of cluster *a* than the centroid of the regular cluster *r*:

(2) $$O_{f}^{ar}=1-\frac{1}{n_{1}+n_{2}}\left(\sum_{x\in C_{a}}\delta_{f}\left(x,\mu_{f}^{r},\mu_{f}^{a}\right)+\sum_{x\in C_{r}}\delta_{f}\left(x,\mu_{f}^{a},\mu_{f}^{r}\right)\right)$$

where

(3) $$\delta_{f}\left(x,Y,Z\right)=(mah_{Y}\left(x_{f},Y\right)<mah_{Z}\left(x_{f},Z\right))$$

$\mu_{f}^{r}$ and $\mu_{f}^{a}$ are the centroid values of feature *f* in the regular cluster *r* and the abnormal comparison cluster *a*, and $n_{1}$ and $n_{2}$ are the number of instances in clusters $C_{a}$ and $C_{r}$, respectively. Variable *x* denotes the individual values being iterated in clusters $C_{a}$ and $C_{r}$. Distances in this context are calculated in Mahalanobis distance rather than Euclidean to account for cluster variance and provide a more meaningful distance between values belonging to different clusters. Dividing by $n_{1}+n_{2}$ provides an overlap ratio describing the extent of intersecting points overlapping on dimension *f*.

Subtracting from 1 constrains the result so that smaller amounts of overlap give larger values for $O_{f}^{ar}$, indicating a greater importance of that feature. Adding 1 in $\left(1+O_{f}^{ar}\right)$ ensures a number greater than 1 and hence ensures the multiplication produces a higher value rather than a lower one, as $O_{f}^{ar}$ is constrained between 0 and 1.

The product of this inverse overlap value modifies the importance score, producing a higher value corresponding with lesser overlap. The higher the overlap between clusters $C_{r}$ and $C_{a}$ over dimension *f*, the less conclusive feature *f* is to the distinction between the two clusters, thus lowering the importance score.

Finally, $\left(1+|\mu_{f}^{r}-\mu_{f}^{a}|\right)$ is the absolute distance between the cluster centroids $\mu_{f}^{r}$ and $\mu_{f}^{a}$ along dimension *f*, augmented by one to ensure that the equation is multiplied by a value ≥1. This constraint ensures a positive correlation between a greater absolute gap between the clusters on the dimension of feature *f* and a greater importance of feature *f* to the distinction between the two clusters.

The corresponding DimWise values for six body regions (head, torso, left arm, left leg, right arm, right leg) are summed to generate 6 scores, which are then processed through a Softmax function to provide each body part’s value as a percentage of the total DimWise score.

This scoring metric forms the basis of the experiment in Section 4 (see Table 3 which demonstrates both the accuracy and relatability of this scoring metric compared to the intuition of healthcare professionals). To provide the model further domain-specific knowledge, DimWise scores are modified with user-defined weights to give greater relative importance to, for example, the leg joints contrasted with the nose joint (the weights used are given in Table A1 of the Appendix A). To gauge the descriptive power of the DimWise metric for describing gait abnormalities, five healthcare professionals across Medicine, Nursing, Occupational Therapy and Social Care were invited to independently rank body regions by their importance in gait assessment based on excerpts from the WeightGait dataset (see Section 4).

While the DimWise score can be used to obtain an approximate description of the detected gait clusters, to improve its interpretability, two additional metrics are introduced as follows:

(1) **Confidence:** the confidence that the clusters represent statistically distinct gait pathologies.
(2) **Severity:** the degree of difference of gait pathologies from regular gait patterns.

The confidence score, for each gait cycle instance, estimates how confident the model is that gait instance *i* belongs to cluster $C^{a}$, as given by:

(4) $$Conf_{a}^{i}=\left(\frac{\max(d_{r}^{i},d_{a}^{i})}{Dist(M_{r},M_{a})}\right)$$

where $d_{r}^{i}$ and $d_{a}^{i}$ are the distances of centroids $M_{r}$ and $M_{a}$ from gait instance *i*, respectively. Confidence is calculated by finding the maximum distance between instance *i* and one of the two centroids (i.e., $\max(d_{r}^{i},d_{a}^{i})$) and dividing by the total distance between clusters *a* and *r* given by $Dist(M_{r},M_{a})$. This formula quantifies the confidence of assigning sample *i* to cluster *a*. “Regular”, in this context, is the cluster that contains the “default” class of each dataset. For example, in WeightGait, this would be the class where the participant has no attached weights.

The confidence metric provides one indication to an end-user as to whether specific instances are likely to be in the cluster they are assigned, whether that cluster is distinct enough to be considered its own class, or whether this specific instance is likely to be influenced by noise. The confidence metric therefore provides a means of evaluating a cluster (through taking the mean confidence of all instances in a cluster), not unlike existing metrics such as Jaccard or Rand indices, with the added benefit of providing greater descriptive power in evaluating how similar any given instance is to one of the clusters relative to others.

To complement the confidence score, a severity score is also introduced. While the confidence score reports the model’s confidence that an instance belongs to a cluster and that the cluster itself is legitimate, the severity score quantifies the *degree* of difference between one cluster of gait instances and the characteristics of “regular” gait. As before, there is a severity score for each gait cycle instance *i*.

The severity score for an instance *i* assigned to cluster *C* is given by:

(5) $$S_{C}^{i}=\frac{d_{r}^{i}}{\frac{1}{k-1}\sum_{j\neq i}d_{r}^{j}}$$

**Figure 3:**
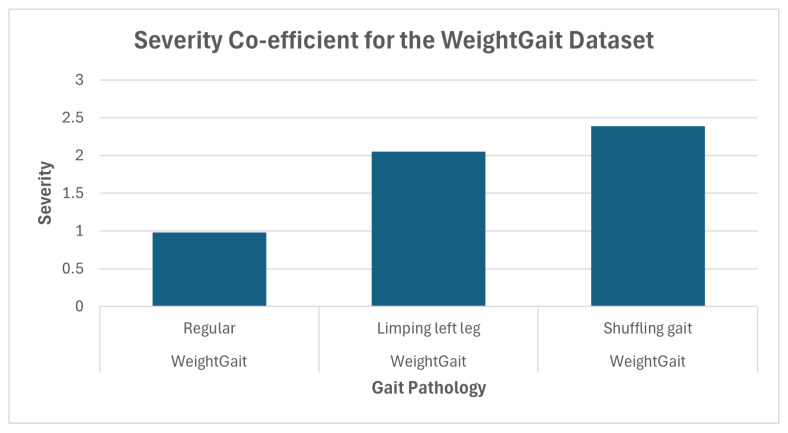
Mean of severity scores for the pathology clusters in the WeightGait dataset.

$d_{r}^{i}$ is the distance from instance *i*’s assigned cluster to the “regular” class centroid. This value is aggregated for all $d_{r}^{k}$ “abnormal” cluster distances. Dividing the distance $d_{r}^{i}$ by this normalization term gives higher severity scores for instances *i* that are relatively further from “regular” than the other abnormal instances. Higher values, especially compared to the average severity scores given by instances in the regular class cluster, give a further general indication that a cluster is indeed composed of a unique gait pathology, and that said pathology is more or less different from regular gait. (Figure 3, as discussed below, demonstrates this point). Observations on how the confidence and severity scores complement the DimWise metric are given in Section 4, including an analysis of how these metrics might be used to interpret results.

## 4. Results

### 4.1. Performance of the Proposed Model

First, we assess the reliability of the embeddings being produced by the trained ST-TAGCN models, which are then further processed using PCA to transform the seven-frame gait cycle representations into single-frame processed embeddings. The proposed method is compared using existing benchmarks across the three datasets, with accuracy being computed as the average classification accuracy across all classes in each dataset, including the “regular” gait class. The ST-TAGCN model used for the proposed embedding is identical in structure to the one used in [28] for which a direct comparison is provided.

Table 2 shows the improved accuracy of the proposed ensemble PCA–neural network embedded approach with K-means clustering, compared to both traditional and state-of-the-art classification methods for gait abnormality assessment. Across all three datasets used as benchmarks, the proposed approach achieves a mean accuracy increase of (4.52%, 4.73%, and 16%), respectively, for the WeightGait [28], Pathological [25] and INIT [15] datasets. To our knowledge, this is the first benchmark for the INIT dataset using the skeletal joint data as opposed to the point-cloud data also provided in the dataset. Reducing the complexity of the feature space, in this case by using the proposed grouped PCA strategy prior to clustering, yields an improvement in describability. This is illustrated by the boost in accuracy across the ablations to the proposed method, as shown in Table 2. The proposed base model outperforms the multi-stream input approaches, and, with the inclusion of PCA, outperforms the best single-stream approach.

### 4.2. Evaluation of the DimWise Metric

While Section 3.3 provides a mathematical justification for the feature importance calculations, the purpose of the next experiment is to demonstrate the practicality of this feature importance formula in application.

Six videos from the WeightGait dataset were shown to the recruited healthcare professionals, two of each gait type, who were asked to rank from 1 to 6 the importance of each joint region in Table 3 to their understanding of the nature of the gait exhibited in each video. To compare results, the means of each participant’s scores across both videos were taken, with the resulting values ranked 1–6 by final value in descending order.

While there is some degree of variability in the comparison, especially towards the lower end of the ranking with the less “important” joints, 33% of DimWise calculations overlap exactly with health professional predictions, rising to 83% when accounting for predictions that are off by a single rank (for example, cases of ranking left and right arms with 4 and 5 versus 5 and 4 for shuffling gait). Full DimWise scores for the other two datasets can be found in Table A1 of Appendix A.

### 4.3. Analysis of the Confidence and Severity Score

The average confidence scores per class across the three datasets calculated using the formula in Equation (4) were 73.6%, 67% and 65.07% for WeightGait, Pathological Gait and INIT, respectively. While these results generally indicate positive confidence, losses in confidence can partly be explained by higher numbers of classes to distinguish from (three in WeightGait compared to nine in INIT) or lower confidence scores for more subtle classes bringing the average down (small and medium pad classes average 64% vs. 72.5% for the ankle weight classes in the INIT dataset). The full table of these results can be seen in Table A2 of Appendix A.

**Figure 4:**
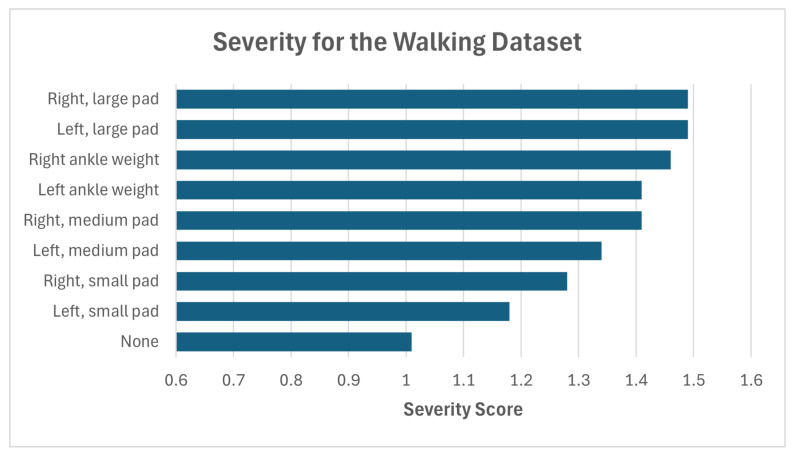
Mean severity scores for the pathology clusters in the Walking Gait dataset.

The results in Figure 3 and Figure 4 show a consistent increase in severity scores relative to the regular cluster in both datasets, illustrating the increasing severity of progressively more noticeable pathologies. The only slight inconsistency is in the case of the INIT dataset, in part likely due to the subtlety of the difference between the pathologies themselves.

The severity score, combined with the DimWise metric, helps build a general picture of each cluster’s gait anomaly without requiring explicit domain knowledge about the abnormality itself. For example, with the limping left leg class in WeightGait, the results show that the difference from the regular gait is significant given the severity score, indicating a notable difference in gait, and the DimWise scores show that the main components of these differences are manifested in the left leg.

## 5. Conclusions

This paper has introduced a more accurate method for pathological gait classification, which includes some medically validated joint-level gait abnormality explainability. Additionally introduced was a method for quantifying feature importance in unknown clusters as a means of producing pathology descriptions. To achieve this, we proposed a novel framework for measuring per-cluster feature importance using the novel DimWise score, alongside severity and confidence scoring mechanisms to reinforce the interpretability of the results. This DimWise metric provides highly explainable outputs without requiring prior labels of pathologies or extensive combinations and comparisons of features, making this method especially useful in a healthcare context where computing power and data are limited.

Using an embedding of a gait sequence generated from an ensemble method of a trained ST-TAGCN model and then condensed through PCA to produce a single-frame representation, the ability to produce improved state-of-the-art gait assessment accuracy is demonstrated. This was validated across three synthetic gait abnormality datasets by 4.52%, 4.73% and 16% when clustering the resultant embeddings using a K-means model. The effectiveness of the three introduced metrics are illustrated, describing the nature of gait pathologies without labels via a ranking of feature importance by body region. The accuracy and intuition of the DimWise metric specifically was verified via comparison of joint ranking with five healthcare professionals.

## 6. Discussion

One limitation of this work is that the proposed method requires prior information regarding how many pathology classes there are, which may not be known in practice and could lead to unstable results. In terms of evaluation, despite the range of opinions across the medical community being in broad agreement with the rankings provided by the model, a larger study to further reinforce these claims would also benefit the technology. To more convincingly test this technology in the field of gait assessment, at-home studies would be a logical next step, attempting to build gait profiles in people over a series of recording periods without explicitly labeled pathologies.

There is a high variability in performance of the DimWise score between the top and bottom ranked joints when compared with rankings provided by experts. This can be explained intuitively as, in the case of limping of the left leg, the torso or individual arms in determining this pathology are unimportant. For both humans or the proposed model, ranking these remaining regions is therefore difficult and irrelevant. The most vital part of the feature importance measurement in this domain would likely be the first two body parts, on which DimWise achieves complete parity with human professionals, bar a single difference in ranking in the left and right legs for the “no abnormality” column.

Compared to prior work [32,33,35], the three metrics introduced in this study offer a more nuanced characterization of gait pathologies, specifically by localizing abnormalities to specific body parts and quantifying deviations relative to an individual’s typical gait. This represents a novel contribution, as existing methods often trade off between model sophistication and interpretability. For instance [32] employs decision trees to generate cluster-based feature descriptions but fails to capture the relative importance of features across clusters. Similarly [33] demonstrates that PCA embeddings can yield high predictive performance for mobility markers, yet the resulting representations are abstract and difficult to communicate in clinical settings. Other studies that focus on richer gait embeddings [35,39] often improve performance but still fall short in providing interpretable insights.

The prime future use of this system would be in precision healthcare; namely, in at-home gait monitoring to provide remote monitoring of individuals either (a) recovering from injury at home or (b) deteriorating from an age-related disease like Alzheimer’s. This would facilitate precision care without needing to sacrifice additional independence that comes with institutionalization in hospital or a care home. Future work includes optimization and improvement of the ensemble embedding pipeline. For example, investigating the degree of geometric information preserved in the embedding from the original nodes and varying the PCA methodology with more components per joint, or the removal of further joints like the eyes and ears to improve performance further.

## Figures and Tables

**Table 1:** The datasets selected for the experiments in Section 4.

Dataset	People	Sequences	Pathology Count	Pathology Types
Pathological Gait Dataset [25]	10	7200	5 + 1 normal	A variety of acted gait pathologies including ataxic, lurching and stiff-legged gait.
Shoe [15]	10	160	8 + 1 normal	8 variations of modified shoe insoles and 4 variations of ankle weights from 1.5 to 3 kg on each leg.
WeightGait Dataset [28]	15	5250	2 + 1 normal	Overlapping pathologies including gait freezing, limping, shuffling and obstacles. Pathologies achieved through a mix of acting and attachable ankle weights.

**Table 2:** Comparison of different models across 3 datasets, including an ablation of the proposed method via the last three model combinations in each section. “Embed” indicates the use of feature embeddings taken from a trained ST-TAGCN, and “PCA” indicates the use of the per-joint PCA strategy described in Section 3.2.

Dataset	Model	Accuracy (%)	F1 + STD
WeightGait (Ours) 3 classes	Logistic Regression	52.77	0.53 ± 0.13
	K-means	32.03	0.34 ± 0.07
	ST-GCN	78.99	0.81 ± 0.32
	ST-JAGCN	89.45	0.83 ± 0.39
	ST-TAGCN	94.38	0.93 ± 0.34
	Embed + K-Means	92.1	0.92 ± 0.6
	**Embed + PCA + K-means (Ours)**	**98.9**	**0.98** ± **0.51**
Pathological [25] 6 classes	Logistic Regression	44.7	0.44 ± 0.1
	K-means	18.7	0.07 ± 0.07
	GRU [25]	93.67	N/A ± N/A
	ST-GCN	92.55	0.89 ± 0.81
	ST-JAGCN	92.47	0.89 ± 0.52
	ST-TAGCN	92.31	0.91 ± 0.67
	ST-TAGCN embed + K-means	77.7	0.77 ± 0.66
	**Embed + PCA + K-means (Ours)**	**98.4**	**0.98** ± **0.45**
INIT [15] 9 classes	Logistic Regression	13.1	0.11 ± 0.09
	K-means	10.2	0.1 ± 0.11
	ST-GCN	75.23	0.72 ± 1.87
	ST-JAGCN	77.03	0.72 ± 1.34
	ST-TAGCN	77.8	0.74 ± 1.04
	ST-TAGCN embed + PCA	58.8	0.55 ± 0.77
	**Embed + PCA + K-means (Ours)**	**93.8**	**0.93** ± **0.68**

**Table 3:** Predicted order of model joint importance compared with the mode of the predictions from five medical professionals for the abnormalities present in the WeightGait dataset. 1 (light green) denotes the most important joint and 6 the least (dark red). The color coding is used to illustrate the similarity of our method’s predictions to the human experts, with clumps of similar colors corresponding to areas of agreement.

Predictor	Abnormality	Head	Torso	Left Arm	Right Arm	Left Leg	Right Leg
ine ML Model	None	4	3	5	6	2	1
Health professional	None	3	5	6	4	1	2
ine							
ine ML Model	Limping left leg	4	3	6	5	1	2
Health professional	Limping left leg	3	6	5	4	1	2
ine							
ine ML Model	Shuffling gait	1	6	4	5	2	3
Health professional	Shuffling gait	1	6	5	4	2	3

**Figure A1:**
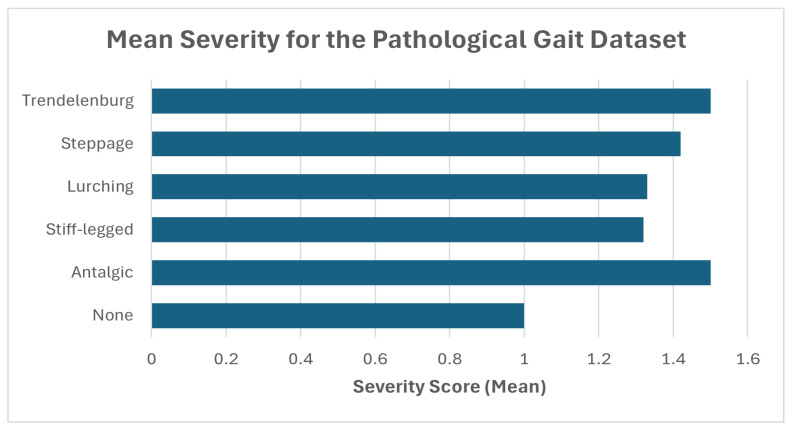
Mean severity scores for the Pathological Gait dataset.

## Data Availability

No new data were created or analyzed in this study.

## Appendix A

The weights in Table A1 were estimated as a means of imbuing the model with human intuition as to the importance of body parts for determining gait, for example, eye joints are intuitively less important than knee joints. The additional benefit for this weighting scheme is to dilute the importance of joints in regions with more joints than others, for example, the head contains more joints than the left leg, so a simple aggregation may provide a faulty picture of genuine importance to the model.

Figure A1 provides the results for mean severity by pathology type for the Pathological Gait dataset, to complement the similar charts in the main paper.

**Table A1:** The weights used to regulate the relative importance of joints in each joint region for the DimWise calculations.

Body Part	No. Joints	Weight
Head	4	0.4
Torso	2	0.8
L.Arm	3	0.3
R.arm	3	0.3
L.Leg	3	0.6
R.Leg	3	0.6

**Table A2:** The ranking of each body part across all pathologies in the datasets. Rankings from 1 to 6 are in brackets, with the outside values denoting the DimWise values. Values are rounded so rows may not precisely sum to 100.

Data	Abnormality	Head	Torso	L. Arm	R. Arm	L. Leg	R. Leg
[28]	Regular	12 (4)	14 (3)	9 (5)	4 (6)	22 (2)	36 (1)
[28]	Limping left leg	10 (4)	14 (3)	6 (6)	9 (5)	39 (1)	19 (2)
[28]	Shuffling gait	22 (2)	10 (6)	12 (5)	14 (4)	23 (1)	16 (3)
[25]	None	12 (4)	15 (3)	8 (5)	6 (6)	27 (2)	30 (1)
[25]	Antalgic	17 (4)	20 (3)	3 (6)	7 (5)	30 (1)	21 (2)
[25]	Stiff-legged	18 (3)	10 (6)	12 (4)	11 (5)	21 (2)	25 (1)
[25]	Lurching	14 (4)	20 (2)	9 (5)	9 (6)	26 (1)	19 (3)
[25]	Steppage	13 (3)	11 (4)	10 (5)	9 (6)	31 (1)	24 (2)
[25]	Trendelenburg gait	13 (4)	15 (3)	10 (6)	11 (5)	23 (2)	26 (1)
[24]	None	17 (2)	16 (3)	11 (6)	12 (5)	13 (4)	28 (1)
[24]	L. foot, small pad	20 (2)	19 (3)	8 (6)	13 (5)	17 (4)	21 (1)
[24]	L. foot, med pad	17 (2)	16 (5)	7 (6)	16 (3)	25 (1)	16 (4)
[24]	L. foot, large pad	15 (4)	19 (2)	9 (6)	16 (3)	25 (1)	14 (5)
[24]	L. foot weight	18 (3)	19 (2)	9 (6)	14 (4)	24 (1)	13 (5)
[24]	R. foot, small pad	13 (4)	14 (3)	14 (2)	12 (5)	9 (6)	35 (1)
[24]	R. foot, med pad	11 (4)	10 (5)	16 (2)	9 (6)	13 (3)	37 (1)
[24]	R. foot, large pad	9 (5)	14 (2)	13 (3)	8 (6)	11 (4)	41 (1)
[24]	R. foot weight	16 (3)	23 (2)	12 (4)	10 (6)	12 (5)	24 (1)

**Table A3:** The mean severity and confidence values by pathology type for the 3 datasets. The means were taken from 10 runs of each dataset.

Dataset	Abnormality	Severity (Mean)	Confidence (Mean)
WeightGait [28]	Regular	0.98	N/A
	Limping left leg	2.05	76.0
	Shuffling gait	2.39	80.21
Pathological Gait [25]	None	1	N/A
	Antalgic	1.5	89.94
	Stiff-legged	1.32	85.86
	Lurching	1.33	75.82
	Steppage	1.42	80.76
	Trendelenburg gait	1.5	80.24
Walking Gait dataset [24]	None	1.01	N/A
	L. foot, small pad	1.18	81.84
	L. foot, medium pad	1.34	88.08
	L. foot, large pad	1.49	87.58
	L. foot ankle weight	1.41	84.36
	R. foot, small pad	1.28	99.4
	R. foot, medium pad	1.56	80.58
	R. foot, large pad	1.49	90.09
	R. foot ankle weight	1.46	83.47

## References

[1] Liu W., Lin X., Chen X., Wang Q., Wang X., Yang B., Cai N., Chen R., Chen G., Lin Y. (2023). Vision-based estimation of mds-updrs scores for quantifying parkinson’s disease tremor severity. Med. Image Anal..

[2] Sepas-Moghaddam A., Etemad A. (2022). Deep gait recognition: A survey. IEEE Trans. Pattern Anal. Mach. Intell..

[3] Tian H., Li H., Jiang W., Ma X., Li X., Wu H., Li Y. (2024). Cross-spatiotemporal graph convolution networks for skeleton-based parkinsonian gait mds-updrs score estimation. IEEE Trans. Neural Syst. Rehabil. Eng..

[4] Zhang J., Lim J., Kim M., Hur S., Chung T. (2023). Wm–stgcn: A novel spatiotemporal modeling method for parkinsonian gait recognition. Sensors.

[5] Wang X., Ellul J., Azzopardi G. (2020). Elderly fall detection systems: A literature survey. Front. Robot. AI.

[6] Yhdego H., Li J., Paolini C., Audette M. Wearable sensor gait analysis of fall detection using attention network. Proceedings of the 2021 IEEE International Conference on Bioinformatics and Biomedicine (BIBM).

[7] Zhao F., Cao Z., Xiao Y., Mao J., Yuan J. (2018). Real-time detection of fall from bed using a single depth camera. IEEE Trans. Autom. Sci. Eng..

[8] Fernandes C., Fonseca L., Ferreira F., Gago M., Costa L., Sousa N., Ferreira C., Gama J., Erlhagen W., Bicho E. Artificial neural networks classification of patients with parkinsonism based on gait. Proceedings of the 2018 IEEE International Conference on Bioinformatics and Biomedicine (BIBM).

[9] Gholami M., Ward R., Mahal R., Mirian M., Yen K., Park K.W., McKeown M.J., Wang Z.J. (2023). Automatic labeling of parkinson’s disease gait videos with weak supervision. Med. Image Anal..

[10] Guo R., Sun J., Zhang C., Qian X. (2022). A self-supervised metric learning framework for the arising-from-chair assessment of parkinsonians with graph convolutional networks. IEEE Trans. Circuits Syst. Video Technol..

[11] Pais B., Buluschek P., DuPasquier G., Nef T., Schütz N., Saner H., Gatica-Perez D., Santschi V. (2020). Evaluation of 1-year in-home monitoring technology by home-dwelling older adults, family caregivers, and nurses. Front. Public Health.

[12] Xue D., Sayana A., Darke E., Shen K., Hsieh J., Luo Z., Li L., Downing N.L., Milstein A., Li F.F. (2018). Vision-based gait analysis for senior care. arXiv.

[13] Jeon S., Lee K.M., Koo S. (2021). Anomalous gait feature classification from 3-d motion capture data. IEEE J. Biomed. Health Inform..

[14] Tao X., Yun Z. (2017). Fall prediction based on biomechanics equilibrium using kinect. Int. J. Distrib. Sens. Netw..

[15] Nguyen T.-N., Huynh H.H., Meunier J. (2018). 3D reconstruction with time-of-flight depth camera and multiple mirrors. IEEE Access.

[16] Yin Z., Jiang Y., Zheng J., Yu H. (2023). Stja-gcn: A multi-branch spatial–temporal joint attention graph convolutional network for abnormal gait recognition. Appl. Sci..

[17] Braspenning A.M., Cranen K., Snaphaan L.J.A.E., Wouters E.J.M. (2022). A multiple stakeholder perspective on the drivers and barriers for the implementation of lifestyle monitoring using infrared sensors to record movements for vulnerable older adults living alone at home: A qualitative study. Int. J. Environ. Res. Public Health.

[18] Hunter I., Elers P., Lockhart C., Guesgen H., Singh A., Whiddett D. (2020). Issues associated with the management and governance of sensor data and information to assist aging in place: Focus group study with health care professionals. JMIR mHealth uHealth.

[19] Itoh S., Tan H., Kudo K., Ogata Y. (2022). Comparison of the mental burden on nursing care providers with and without mat-type sleep state sensors at a nursing home in tokyo, japan: Quasi-experimental study. JMIR Aging.

[20] Yu B., Yin H., Zhu Z. (2017). Spatio-temporal graph convolutional networks: A deep learning framework for traffic forecasting. arXiv.

[21] Guo R., Shao X., Zhang C., Qian X. (2021). Multi-scale sparse graph convolutional network for the assessment of parkinsonian gait. IEEE Trans. Multimed..

[22] Filtjens B., Ginis P., Nieuwboer A., Slaets P., Vanrumste B. (2022). Automated freezing of gait assessment with marker-based motion capture and multi-stage spatial-temporal graph convolutional neural networks. J. Neuroeng. Rehabil..

[23] Sabo A., Mehdizadeh S., Iaboni A., Taati B. (2022). Estimating parkinsonism severity in natural gait videos of older adults with dementia. IEEE J. Biomed. Health Inform..

[24] Ortells J., Herrero-Ezquerro M.T., Mollineda R.A. (2018). Vision-based gait impairment analysis for aided diagnosis. Med. Biol. Eng. Comput..

[25] Jun K., Lee Y., Lee S., Lee D., Kim M.S. (2020). Pathological gait classification using kinect v2 and gated recurrent neural networks. IEEE Access.

[26] Wang L., Chen J., Chen Z., Liu Y., Yang H. (2022). Multi-stream part-fused graph convolutional networks for skeleton-based gait recognition. Connect. Sci..

[27] Yu S., Tan D., Tan T. A framework for evaluating the effect of view angle, clothing and carrying condition on gait recognition. Proceedings of the 18th International Conference on Pattern Recognition (ICPR’06).

[28] Lochhead C., Fisher R.B. (2025). Lightweight human gait anomaly assessment using single-stream uniform temporal attention. Comput. Biol. Med..

[29] Lundberg S.M., Lee S. (2017). A unified approach to interpreting model predictions. Adv. Neural Inf. Process. Syst..

[30] Ribeiro M.T., Singh S., Guestrin C. “why should i trust you?” explaining the predictions of any classifier. Proceedings of the 22nd ACM SIGKDD International Conference on Knowledge Discovery and Data Mining.

[31] Moshkovitz M., Dasgupta S., Rashtchian C., Frost N. Explainable k-means and k-medians clustering. Proceedings of the International Conference on Machine Learning, PMLR 2020.

[32] Laber E., Murtinho L., Oliveira F. (2023). Shallow decision trees for explainable k-means clustering. Pattern Recognit..

[33] Young-Shand K.L., Roy P.C., Dunbar M.J., Abidi S.S.R., Wilson J.L.A. (2023). Gait biomechanics phenotypes among total knee arthroplasty candidates by machine learning cluster analysis. J. Orthop. Res..

[34] Zhang Z., Tran L., Yin X., Atoum Y., Liu X., Wan J., Wang N. Gait recognition via disentangled representation learning. Proceedings of the IEEE/CVF Conference on Computer Vision and Pattern Recognition.

[35] Wang G., Yuan Y., Chen X., Li J., Zhou X. Learning discriminative features with multiple granularities for person re-identification. Proceedings of the 26th ACM international conference on Multimedia.

[36] Duvenaud D.K., Maclaurin D., Iparraguirre J., Bombarell R., Hirzel T., Aspuru-Guzik A., Adams R.P. (2015). Convolutional networks on graphs for learning molecular fingerprints. Adv. Neural Inf. Process. Syst..

[37] Lu Z., Du P., Nie J. Vgcn-bert: Augmenting bert with graph embedding for text classification. Proceedings of the Advances in Information Retrieval: 42nd European Conference on IR Research, ECIR 2020.

[38] Hofmann M., Geiger J., Bachmann S., Schuller B., Rigoll G. (2014). The tum gait from audio, image and depth (gaid) database: Multimodal recognition of subjects and traits. J. Vis. Commun. Image Represent..

[39] Ismaili O.A., Lemaire V., Cornuéjols A. A supervised methodology to measure the variables contribution to a clustering. Proceedings of the Neural Information Processing: 21st International Conference, ICONIP 2014.

[40] Liu Y., Li Z., Xiong H., Gao X., Wu J. Understanding of internal clustering validation measures. Proceedings of the 2010 IEEE International Conference on Data Mining.

